# Interplay of Ecological Opportunities and Functional Traits Drives the Evolution and Diversification of Millettiod Legumes (Fabaceae)

**DOI:** 10.3390/genes13122220

**Published:** 2022-11-27

**Authors:** Dongzhu Jiang, Linzheng Liao, Haitao Xing, Zhidan Chen, Xuemei Luo, Hong-Lei Li

**Affiliations:** College of Landscape Architecture and Life Science/Institute of Special Plants, Chongqing University of Arts and Sciences, Chongqing 402168, China

**Keywords:** diversity, the Millettiod legumes, phylogeny, ecological opportunities, Miocene

## Abstract

Understanding the striking diversity of the angiosperms is a paramount issue in biology and of interest to biologists. The Millettiod legumes is one of the most hyper-diverse groups of the legume family, containing many economically important medicine, furniture and craft species. In the present study, we explore how the interplay of past climate change, ecological opportunities and functional traits’ evolution may have triggered diversification of the Millettiod legumes. Using a comprehensive species-level phylogeny from three plastid markers, we estimate divergence times, infer habit shifts, test the phylogenetic and temporal diversification heterogeneity, and reconstruct ancestral biogeographical ranges. We found that three dramatic accumulations of the Millettiod legumes occurred during the Miocene. The rapid diversification of the Millettiod legumes in the Miocene was driven by ecological opportunities created by the emergence of new niches and range expansion. Additionally, habit shifts and the switch between biomes might have facilitated the rapid diversification of the Millettiod legumes. The Millettiod legumes provide an excellent case for supporting the idea that the interplay of functional traits, biomes, and climatic and geographic factors drives evolutionary success.

## 1. Introduction

Angiosperms are the most prominent life form across global ecosystems, and exhibit a spectacular range of morphological and ecological diversity on the Earth [1]. However, the species diversity across the Tree of Life of the angiosperms is uneven. Understanding the striking diversity of the angiosperms is a paramount issue in biology and of interest to biologists [1,2,3]. Previous studies found that the rapid radiations of many angiosperm groups was driven by both biological (intrinsic) and environmental (extrinsic) factors [4,5,6,7]; however, the effects of functional traits, the biome, and climatic and geographic factors on plant diversification are largely unknown. 

The Millettiod legumes is one of the most hyper-diverse groups in the legume family. This clade is composed of five tribes (Millettieae, Abreae, Phaseoleae, Desmodieae and Psoraleeae), including more than 2600 described species with a global distribution [8,9]. The Millettiod legumes contain many economically important species, which contribute enormously to the word’s economy through food and vegetables, pharmaceuticals and medicines, furniture and crafts, chemicals and fertilizers, as well as horticulture [9]. The main dietary legumes include pigeon pea (*Cajanus cajan* Millsp.), soybean (*Glycine max* Merr.), cowpea (*Vigna unguiculata* Walp.), and bean (*Phaseolus*). Vetches (*Vicia amoena* Fisch.) and tropical kudzu (*Pueraria phaseoloides* Benth.) were important pastures used for forage. Species such as *Desmodium*, *Macoptilium* and *Centrosema* are used for the purpose of improving tropical pasture systems [10]. Showy-flowered species of *Mucuna* and *Strongylodon* are very popular in tropical gardens. Species of *Erythrina* are important woody tree legumes in forestry. Multipurpose shrubs, such as *Butea* and *Millettia*, are regional favorites as ornaments and for shade [9]. 

The Millettiod legumes show great diversity in their morphological and habit characteristics from ephemeral herbs and herbaceous climbers to shrubs, woody lianas and trees. The terminal taxa of the Millettiod legumes grow in various habitats such as succulent, grass, rainforest and temperate, which are in accordance with major zonobiomes [9,11]. The crown age of the Millettiod legumes is 44.1 Ma to 53 Ma. Global climate and tectomic changes since the Eocene may have triggered the diversification of many angiosperm lineages [12,13,14,15,16]. Moreover, the diversification of *Psoraleeae* is assumed to be a result of global climate change during the Pleistocene [17]. The evolution of phaseoloid legumes may be due to habit shifts and dispersal events along with the global climate changes since the Oligocene [18]. However, it remains puzzling and unexplored how the evolution of habits fostered diversification in the Millettiod legumes and how ecological forces have been regulating cladogenesis in different geographical areas.

A well-resolved phylogeny of Millettiod legumes is paramount for understanding the aspects of the temporal and biogeographical patterns of diversification. Some previous molecular phylogenetic studies have made progress in the delimitation of the Millettiod legumes based on nuclear ribosomal *ITS* or a few plastid loci [18,19,20,21,22]. These researches generated a well-resolved phylogenetic framework of the Millettiod legumes at tribal levels [3,23]. However, less than 40% of the generic diversity of the Millettiod legumes was sampled in previous studies. Therefore, a well-resolved phylogeny with dense taxon sampling at species level is needed to understand the evolutionary dynamics of the Millettiod legumes. Oyebanji et al. proved that plastome is an effective marker for resolving the evolutionary relationships of the Millettiod legumes [23]. In the past decades, plastome data of the Millettiod legumes increased rapidly along with previous studies [19,20,21,22,24,25,26,27]. The data accumulation of the Millettiod legumes provides an opportunity to reconstruct the phylogeny of this clade with comprehensive taxon sampling.

In the present study, we first reconstruct a species-level phylogeny for the phaseoloid legumes using three plastid loci with a more extensive generic sampling than in any previous studies. Based on the improved phylogenetic framework, we then explore how the interplay of past climate change, geographical expansion and habit shifts may have triggered diversification of the Millettiod legumes.

## 2. Material and Methods

### 2.1. Taxon and Nuclear Marker Sampling

Three plastid regions (*rbcL*, *matK* and *trnL-F*) were selected as DNA markers for the phylogenetic analyses of the Millettiod legumes. The combination of these three plastid regions has been proved to have high-efficiency in resolving intergeneric and interspecific relationships. To obtain dense sampling of the Millettiod legumes, all available nucleotide sequences of these three markers from GenBank were collected firstly. Then, sequences were filtered following the principles: (1) For each taxon, the same species and DNA sample across three plastid markers were kept in priority, but some composite accessions were necessary to represent genera. (2) When multiple sequences were available for each marker, the longest sequence was retained. (3) In the scenario of multiple sequences with the same length, one sequence was randomly selected. (4) In a genus, when one marker occurred in species different with other markers, the species with faster evolved marker were kept following the order: *trnL-F, rbcL*, *matK.* All the works above were completed by using a series of R scripts written by our group. Most of the DNA sequences have been used in previously published studies [19,20,21,22,24,25,26,27]. Species names and GenBank accession numbers are listed in Table S1.

### 2.2. Alignment and Phylogeny

For the two DNA coding genes (*rbcL* and *matK*), sequences were aligned directly in the program MUSCLE with the default settings [28]. Then the aligned matrix was manually adjusted using BioEdit version 5.0.9 (Hall, T.A., California, USA) [29]. For the plastid region *trnL-F*, sequences were aligned using a two-step strategy. First, sequences were divided into clusters according to taxonomic unit and aligned in the program MUSCLE under default high accuracy parameters with subsequently manual adjustment. Then, the matrix of each cluster was aligned using the profile–profile alignment algorithm in MUSCLE and final adjustments were made to the alignments using the refinement algorithm in MUSCLE. Alignment of each plastid region was manually trimmed for quality and maximum coverage. The final aligned matrix contains 429 OTUs.

To check the conflicts among three plastid regions, the initial phylogenetic analysis for each marker was carried out using maximum likelihood (ML) method implemented in the program RAxML version 7.6.6 (Stamatakis, A., Lausanne, Switzerland) [30]. After checking for significant conflict among different nodes (bootstrap support value exceeding 70%), all data from different markers were combined for further analyses. We used the ML method to construct the phylogeny of the Millettiod legumes based on the combined data. The ML analysis was performed using RAxML with the following options: three data partitions (*rbcL*, *matK* and *trnL-F*), GTR + I + G nucleotide substitution model, and gaps treated as missing data. A standard bootstrap analysis with 1000 bootstrap iterations was used to estimate the branch support. The program was run on the CIPRES network [31]. 

### 2.3. Molecular Dating

To date the branching events within the Millettiod legumes, a Bayesian MCMC approach as implemented in BEAST version 1.8.0 (Drummond, A.J., Auckland, Zealand) was used based on the combined dataset of 429 terminals [32]. We selected 7 calibration points from the result of Lavin et al. [33]. These calibration points could be confidently assigned to clades and nodes represented in our dataset (Table S2). A normal distribution was used for all 7 calibration points. The standard deviation was set to contain the lower and higher boundaries of the 95% highest posterior density values. MCMC searches were run for 500,000,000 generations, sampled every 5000 generations. Tracer v1.5 was used to monitor appropriate burn-in and the adequate effective sample sizes of the posterior distribution (>200). The maximum clade credibility tree was computed by TreeAnnotator v1.7.5 in BEAST software package [34]. BEAST analyses were performed in the CIPRES Web Portal 3.1 (Miller, M.A., California, USA) [31].

### 2.4. Trait Evolution Analyses

To clarify the trait evolutionary history of the Millettiod legumes, the habit and biome information were collected (Table S2). The habit information of the Millettiod legumes was collected from International Legume Database & Information Service (ILDIS). Two habit states were coded, herbaceous (including herbs and herbaceous climbing vines) vs. woody (including trees, woody climbers and shrubs) (Table S1). According to the geographic distributions and the biome patterns of Lewis et al. [9], five biome types were coded: (1) succulent (S), including semi-arid, non-fire-adapted, succulent-rich and grass-poor, dry tropical forest, thicket and bushland biome; (2) grass (G), comprising a fire-adapted, succulent-poor and grass-rich, seasonally dry tropical forest, woodland and savanna biome; (3) rainforest (R), comprising the equatorial tropics (wet forests) worldwide; (4) temperate north (TN), comprising the Mediterranean, warm and cold temperate regions of the North Hemisphere; and (5) temperate south (TS), comprising the area except for the first three biomes (Table S1). The parsimony method implemented in Mesquite v2.74 was used to reconstruct the trait evolution of the Millettiod legumes [35]. The maximum clade credibility tree obtained from the Bayesian analysis was used as input tree.

### 2.5. Diversification Analyses

To visualize the temporal dynamics of the diversification rates in the Millettiod legumes, we constructed the semilogarithmic lineage-through-time (LTT) plots using the R package APE v2.5-1 [36]. In total, 1000 dated phylogenies obtained from the Bayesian analyses were randomly selected and used to generate semilogarithmic LTT plots. To verify the rapid shifts in diversification rates at any specified time, the RC statistic was performed using the R package GEIGER v1.3-1 [37]. We used 11 models including pureBirth, bd, DDX, DDL, SPVAR, EXVAR, BOTHVAR, yule2rate, yule3rate, yule4rate and yule5rate to assess the significant rate changes. A diversification rate shift was confirmed by the criterion that more or fewer descendants than expected under the constant rate model happen in lineages. 

### 2.6. Biogeographical Analyses

In order to infer the possible ancestral ranges of the Millettiod legumes, a Bayes-DIVA analysis [38] was conducted using the software package RASP [39]. Bayes-DIVA method is a popular method with the advantages of minimizing the phylogenetic uncertainties and generating credibility support values for alternative phylogenetic relationships [38,39]. We used 1000 trees from the BEAST output as a “trees file”. The maximum clade credibility tree was used as a final representative tree. With the aim of predicating the ancestral areas of nodes deeper down into the tree, biogeographical analyses were run on continental spatial scale at species level. According to Lewies et al. [9] and the ILDIS database, nine geographic regions were coded: A, Africa; B, Asia; C, Australia; D, Indian Ocean area, including islands in the Indian Ocean region and Madagascar; E, Pacific, including islands in the Pacific; F, North America; H, Central America; I, Europe; K, South America (Table S1). The “maxareas” was constrained to 3 when constructing the ancestral areas of the Millettiod legumes.

## 3. Results

### 3.1. Phylogeny of the Millettiod Legumes

The aligned matrix of the combined three-marker dataset consists of 4728 characters with 2290 variable and 1690 parsimony-informative sites. The tree generated by the maximum likelihood (ML) analysis (Figure S1) was highly consistent with those retrieved from the Bayesian inference (BI) analysis (Figure S2), except for some weakly supported nodes (BS < 70%). The Millettiod legumes are strongly supported as monophyletic (BS 100%, PP 1.0). Within the Millettiod legumes, five major clades were identified: Basal millettioid (BS 94%, PP 1.0), Clitoriinae (BS 59%, PP 0.87), Disynstemon + (Abreae + (Diocleinae + core Millettieae)) (BS 81%, PP 1.0), *Platycyamus* + *Dewevrea* (BS 91%, PP 1.0), and the phaseoloid clade (BS 98%, PP 1.0). Within the phaseoloid clade, Desmodieae, Cajaninae and Phaseolinae are monophyletic with high support. Psoraleeae is monophyletic and nested in Glycininae.

### 3.2. Divergence-Time Estimates of the Millettiod Legumes

**Figure 1:**
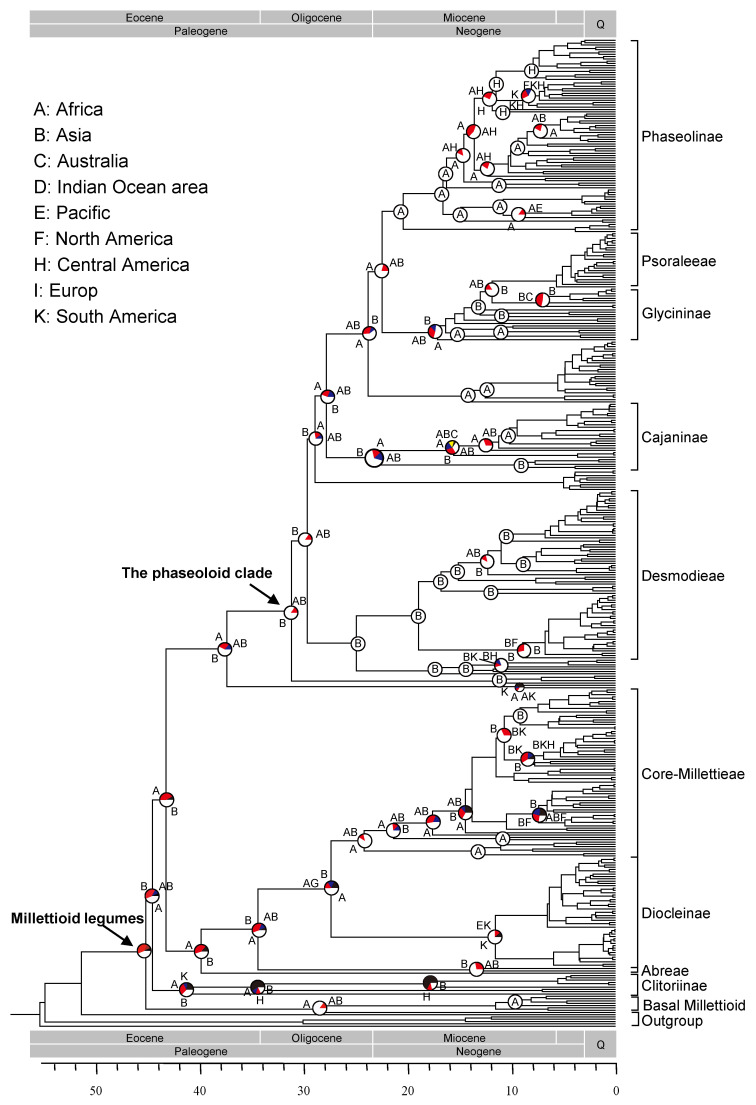
Combined chronogram and the summary of biogeographic analysis of the Millettiod legumes. Dating analysis was performed using BEAST software. The topology corresponds to the majority rule consensus tree of the Bayesian stationary sample. Large pie charts show the relative probabilities of alternative ancestral distributions obtained by statistical dispersal–vicariance analysis (white > red > blue > yellow); areas (frequencies < 0.1) are collectively given with black color.

Based on our analyses, the Millettiod legumes diversified at 45.1 Ma (42.3–47.3 Ma, HPD) (Figure 1 and Figure S3). The second lineage separated from the remaining Millettiod legumes at 44.5 Ma (40.1–45.6 Ma, HPD). The tribe Abreae split with its sister lineage at 34.3 Ma (32.2–38.2 Ma, HPD). The divergence time between core Millettieae and Diocleinae was estimated to be 27.3 Ma (26.4–35.4 Ma, HPD). The steam age of the phaseoloid clade was 37.3 Ma (36.1–41.4 Ma, HPD), and the crow age was 31.1 Ma (28.8–33.5 Ma, HPD). Desmodieae diverged from its close relatives at 24.8 Ma (23.7–30.0 Ma, HPD). Psoraleeae separated from *Glycine* at 11.8 Ma (8.8–14.4 Ma, HPD).

### 3.3. Trait Evolution

**Figure 2:**
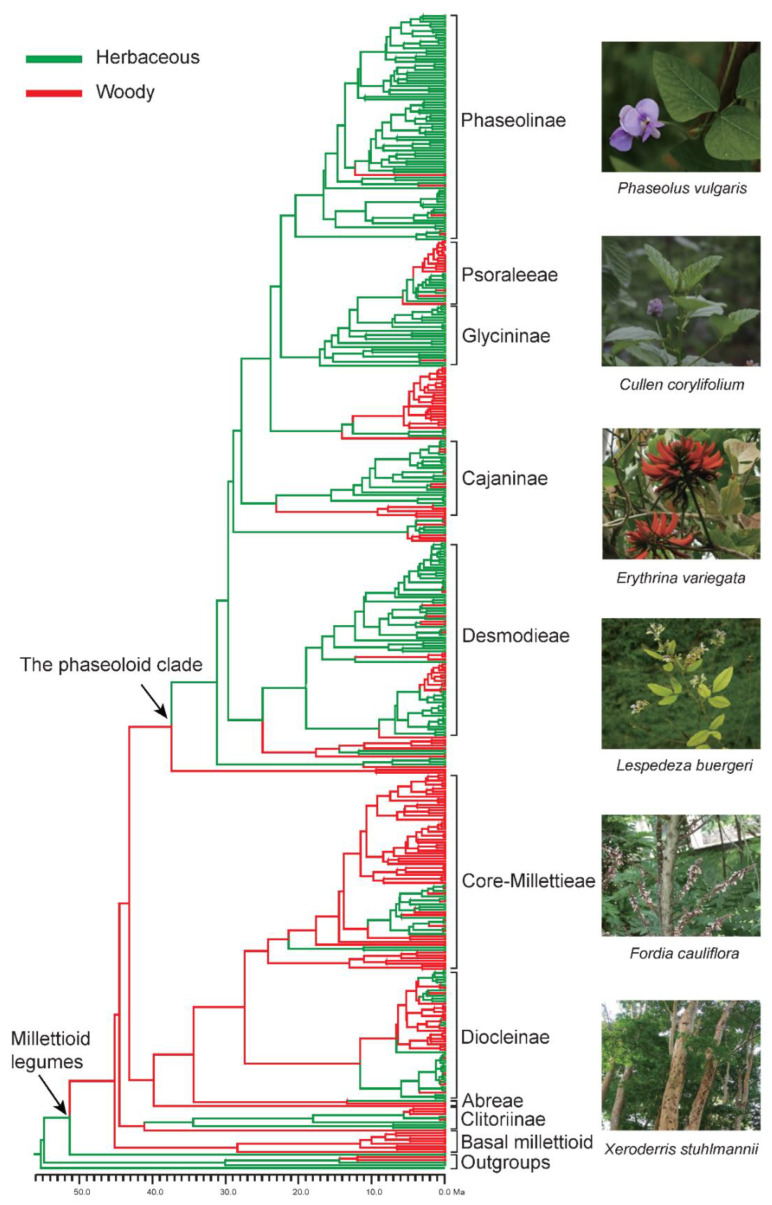
Habit shift analysis of the Millettiod legumes. Green represents herbaceous; red represents woody. Images are representative species from the Millettiod legumes.

**Figure 3:**
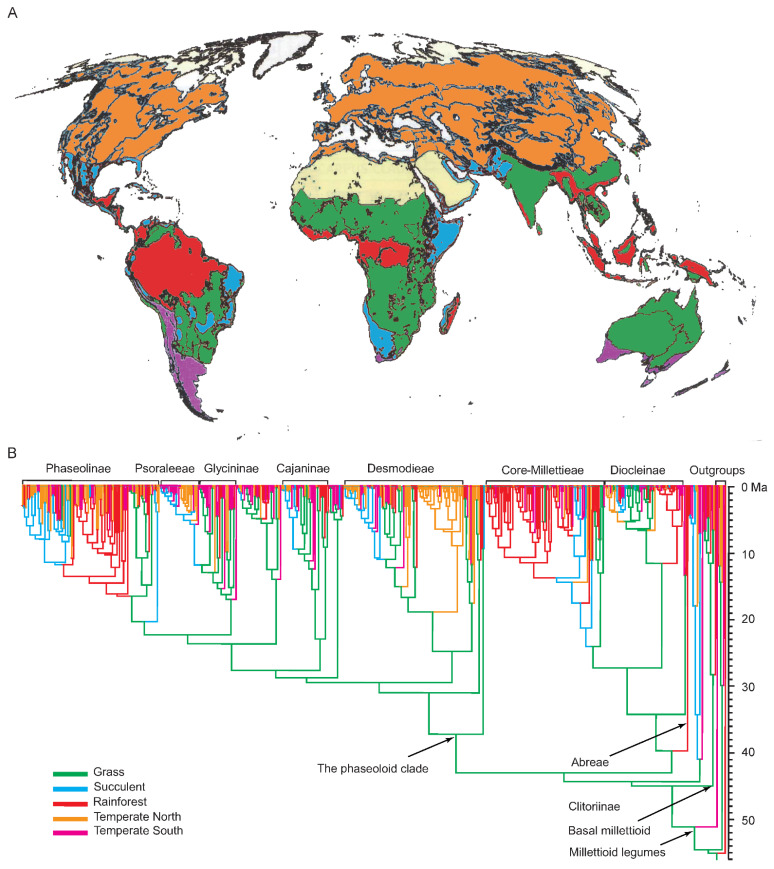
Biome evolutionary history of the Millettiod legumes. (**A**) World map of biome (edited from Lewis et al., 2005 [9]). (**B**) Ancestral biome state reconstruction of the Millettiod legumes using parsimony method.

Results of the ancestral habit state reconstruction of the Millettiod legumes are shown in Figure 2. The ancestral state of the Millettiod legumes was woody. Multiple transitions between woody and herbaceous occurred during the evolutionary history of the Millettiod legumes. Our results showed that switching frequency from woody to herbaceous was higher than that from herbaceous. The switching frequency from woody to herbaceous was 52, whereas the switching frequency from herbaceous to woody was 25. In our examining transitions analyses among biomes, the common ancestor of the Millettiod legumes was most likely distributed in a grass biome (Figure 3). The ancestor of core Millettieae similarly may have occupied a grass biome with subsequent shifts into the other biome types. The ancestor of the phaseoloid clade was also distributed in a grass biome. Multiple shifts between biome types in different clades were detected since the Miocene.

### 3.4. Reconstruction of Historical Biogeography

The results of biogeographic reconstruction are summarized in Figure 1 and Figure S4. The most recent common ancestor (MRCA) of the Millettiod legumes was equivocal, either in Africa (*p* = 0.44), Asia (*p* = 0.39) or the other regions (*p* = 0.17). Multiple colonization events occurred independently during the evolutionary history of the Millettiod legumes. The MRCA of core Millettieae was Africa (*p* = 0.88), and dispersed from Africa to Asia, North America, Central America and South America multiple times. The MRCA of the phaseoloid legumes occurred in Asia (*p* = 0.84), with a number of movements into Africa, Australia and Central America. Among all of the nine geographic regions, Africa and Asia harbored higher frequency of lineage emigration (185 and 204 times, respectively) than immigration (73 and 90 times, respectively). Conversely, the frequency of lineage emigration in Australia, the Indian Ocean area and Pacific harbor was lower with the numbers being 17, 10 and 17.5, respectively, whereas the frequencies of lineage immigration were 81, 87 and 69, respectively.

### 3.5. Diversification Rates

**Figure 4:**
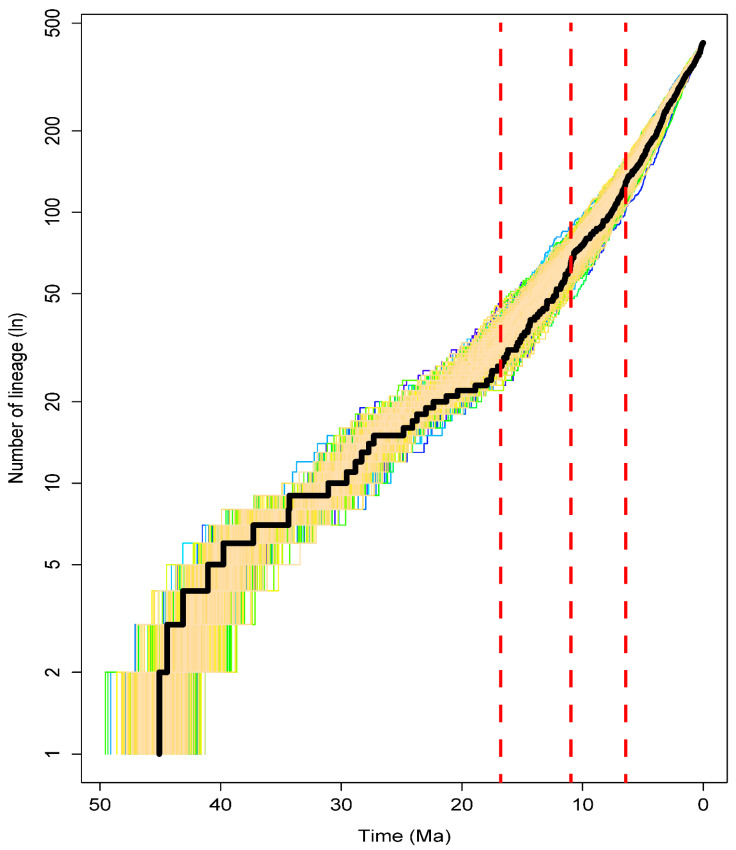
Lineage-through-time (LTT) plots of the Millettiod legumes. Dotted lines in red indicate the rapid increases in diversification rate.

Our results of the semilogarithmic lineage-through-time (LTT) showed that the Millettiod legumes experienced a high diversification rate in the Miocene (Figure 4). When testing the significant rate changes based on 11 models, the AIC values of seven constant rate models, including pureBirth, bd, DDX, DDL, SPVAR, EXVAR and BOTHVAR, were found to be higher than the other four and were excluded (Table 1). Among the variable rate models, the AIC value of yule5rate was the lowest and was selected as the best fit model for the diversification rate analyses of the Millettiod legumes. Three rapid increases in diversification rate were found in the Millettiod legumes: the first was at 17.78 Ma with a rate change from 0.07 to 0.13 (sp Myr^−1^); the second was at 11.57 Ma, with a rate change from 0.13 to 0.29 (sp Myr^−1^); the third was at 8.46 Ma, with a rate change from 0.10 to 0.19 (sp Myr^−1^).

## 4. Discussion

Reliable molecular dating is an essential precondition for revealing the diversification patterns of plant lineages. Phylogenetic analyses of the combined three-region DNA dataset generated a well-supported evolutionary framework of the Millettiod legumes (Figure 1, Figures S1 and S2). The relationships among the main lineages of the Millettiod legumes were in accordance with previous studies [3,18,21,22,23]. Based on our time estimates, the crown age of the Millettiod legumes was 45.1 Ma (42.3–47.3 Ma, HPD; Figure 1 and Figure S3), which corresponded to previous studies [1,33]. The crown age of the phaseoloid legumes was 31.1 Ma (28.8–33.5 Ma, HPD), which was consistent with the estimations of many previous studies [18,26,33]. Our result showed the crown age of Psoraleeae was 5.6 Ma (4.29–8.4 Ma, HPD), which was in accordance with the result of Egan and Crandall [17]. All these results indicated that the divergence times of the Millettiod legumes in the present study were credible.

Our biogeographic reconstruction and dating analyses indicated the Millettiod legumes occurred in Africa (*p* = 0.44) or Asia (*p* = 0.39) in the early Eocene. The LTT plot indicated that a dramatic burst of diversification of the Millettiod legumes occurred during the Miocene (c. 18–8 Ma; Figure 4). Three dramatic accumulations of the Millettiod legumes were detected at 17.8 Ma, 11.6 Ma and 8.5 Ma (Table 1). In accordance with the diversification patterns, high frequent migrations of the Millettiod legumes did not occur until the Miocene after its origin with the exception of some dispersal events into Central America and South America (clade Clitoriinae). Multiple colonization events seem to have occurred within a 10-million-year time window (c. 18–8 Ma, Figure 1). The ancestor of the Diocleinae experienced dispersal events into Central America, South America and the Pacific. There were at least three dispersal events into Central America and South America for the core Millettieae, and two such movements into the Indian Ocean area (Figure 1 and Figure S4). Additionally, the Phaseoloids clade experienced multiple dispersal events from Africa to Central America. In the Miocene, the Qinghai–Tibetan Plateau experienced multiple stages of uplift with a rapid uplift at 10–7 Ma [40,41,42,43,44]. The African plate collided with the Eurasian at ca. 18–17 Ma [45]. In the southern hemisphere, the thrust of the South American plate against the Pacific plate led to the rise of the Andes mountains [46]. These geological events fragmented the ecosystems and subsequently opened many new niches [47,48], and forced the rapid diversification of many plant lineages [16,49,50,51,52,53]. Thus, our finding suggests that the rapid diversification of the Millettiod legumes in the Miocene was driven by ecological opportunities created by the emergence of new niches and range expansion.

In the Miocene, the global climate tended to be cooler, drier and more seasonal [54,55,56]. Both Asia and America experienced an arid period of marked aridity due to orogenetic changes [57,58,59,60]. The African plate also experienced an analogous arid period of aridity due to the closure of the Tethys Sea [45,61]. Our trait evolution analyses showed that a larger number of lineages in the 10-million-year time window (c. 18–8 Ma) were herbaceous and inhabited a grass biome. The grass biome generally has a unimodal rainfall pattern, which comprises a fire-adapted, succulent-poor and grass-rich, seasonally dry tropical forest, woodland and savanna [9,11]. The extensive aridity could have promoted the diversification of some groups inhabiting dry regions, such as *Bursera* [62] and the *Potentilla* [63]. Herbs could have produced higher per-year mutation rates on the basis of a shorter generation time, consequentially contributing to the adaptation of herbaceous lineages to dry climates [64,65,66]. Additionally, multiple shifts from the grass biome to other biomes, especially the rainforest biome, were found in the Miocene. The interdigitation of the grass and the rainforest biome favors a “refuge” interpretation of allopatric divergence, which was caused by the contraction and expansion of dry-adapted and wet vegetation [67]. Taxa adapted to different biomes are always pioneer species, which can take advantage most effectively of post-disturbance conditions [9]. Thus, the expansion of arid lands due to orogenetic and climatic changes increased the arid niche space, and the switch between biomes might have facilitated the rapid diversification of the Millettiod legumes during the Miocene.

## Figures and Tables

**Table 1:** Results of diversification rate model-fitting test with parameters estimated under each model. “R” indicates the speciation rate, “St” indicates where the speciation rate shifts.

Model	AIC	Likelihood	R1	R2	R3	R4	R5	St1	St2	St3	St4
pureBirth	−1910.211	956.1055	0.16607	-	-	-	-	-	-	-	-
bd	−1928.547	966.2735	0.10794	-	-	-	-	-	-	-	-
DDX	−1930.799	967.3994	0.05211	-	-	-	-	-	-	-	-
DDL	−1908.208	956.1041	0.16608	-	-	-	-	-	-	-	-
SPVAR	−1926.028	966.0142	0.24839	-	-	-	-	-	-	-	-
EXVAR	−1926.544	966.2718	0.23662	-	-	-	-	-	-	-	-
BOTHVAR	−1924.329	966.1644	0.24951	-	-	-	-	-	-	-	-
yule2rate	−1929.337	967.6686	0.16650	0.10857	-	-	-	0.04	-	-	-
yule3rate	−1932.285	971.1423	0.07166	0.14114	0.19292	-	-	17.78	7.12	-	-
yule4rate	−1931.881	972.9404	0.09826	0.28943	0.09592	0.19	-	11.57	10.68	8.46	-
yule5rate	−1932.951	975.4753	0.07166	0.13342	0.28943	0.09592	0.19	17.78	11.57	10.68	8.46

## Supplementary Materials

The following supporting information can be downloaded at: https://www.mdpi.com/article/10.3390/genes13122220/s1, Figure S1. Phylogenetic relationships of the phaseoloid legumes using maximum-likelihood (ML) method; Figure S2. Phylogenetic relationships of the phaseoloid legumes using Bayesian inference (BI) method; Figure S3. Chronogram for the Millettiod legumes obtained under a Bayesian relaxed clock; Figure S4. Biogeographic analysis of the Millettiod legumes by Statistical Dispersal-Vicariance Analysis (S-DIVA).; Table S1. Taxa, GenBank accession numbers, Life form code and Biome code for samples included in this study; Table S2. Calibrations used in this study. Node number followed Lavin et al., 2005.
