# Supplementary figures and images for: Interplay of Ecological Opportunities and Functional Traits Drives the Evolution and Diversification of Millettiod Legumes (Fabaceae)
Source: Genes (Basel). 2022 Nov 27;13(12):2220. doi: 10.3390/genes13122220 (PMC9777679; doi:10.3390/genes13122220)

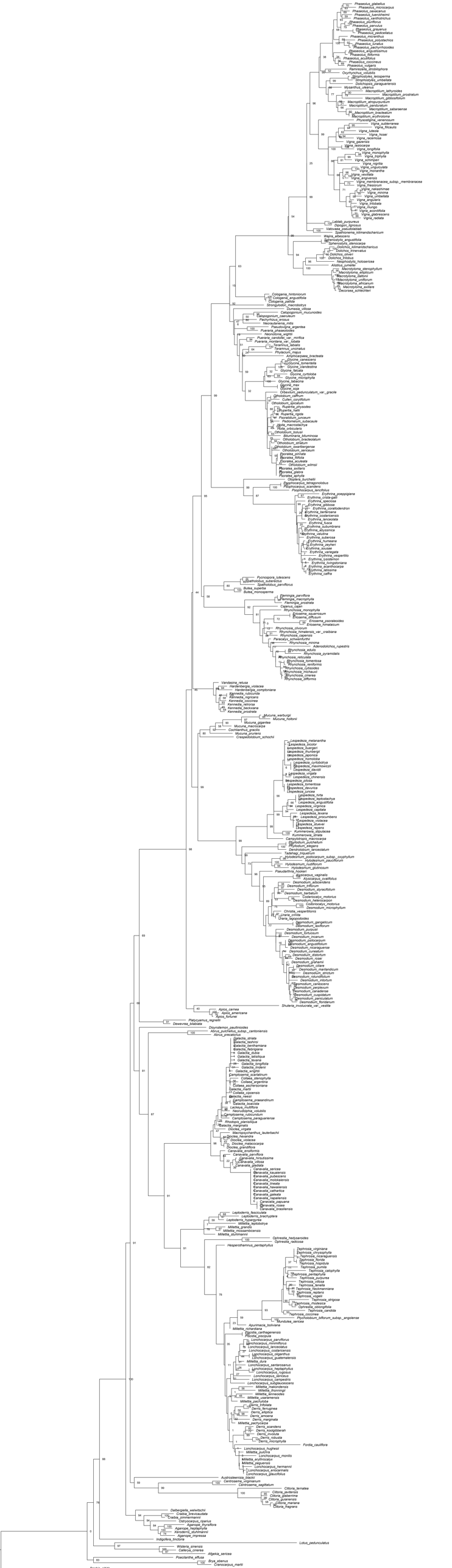

Supplement: Supplementary file 1 [file genes-13-02220-s001.zip › Figure S1. Phylogenetic relationships of the phaseoloid legumes using maximum-likelihood (ML) method.pdf]

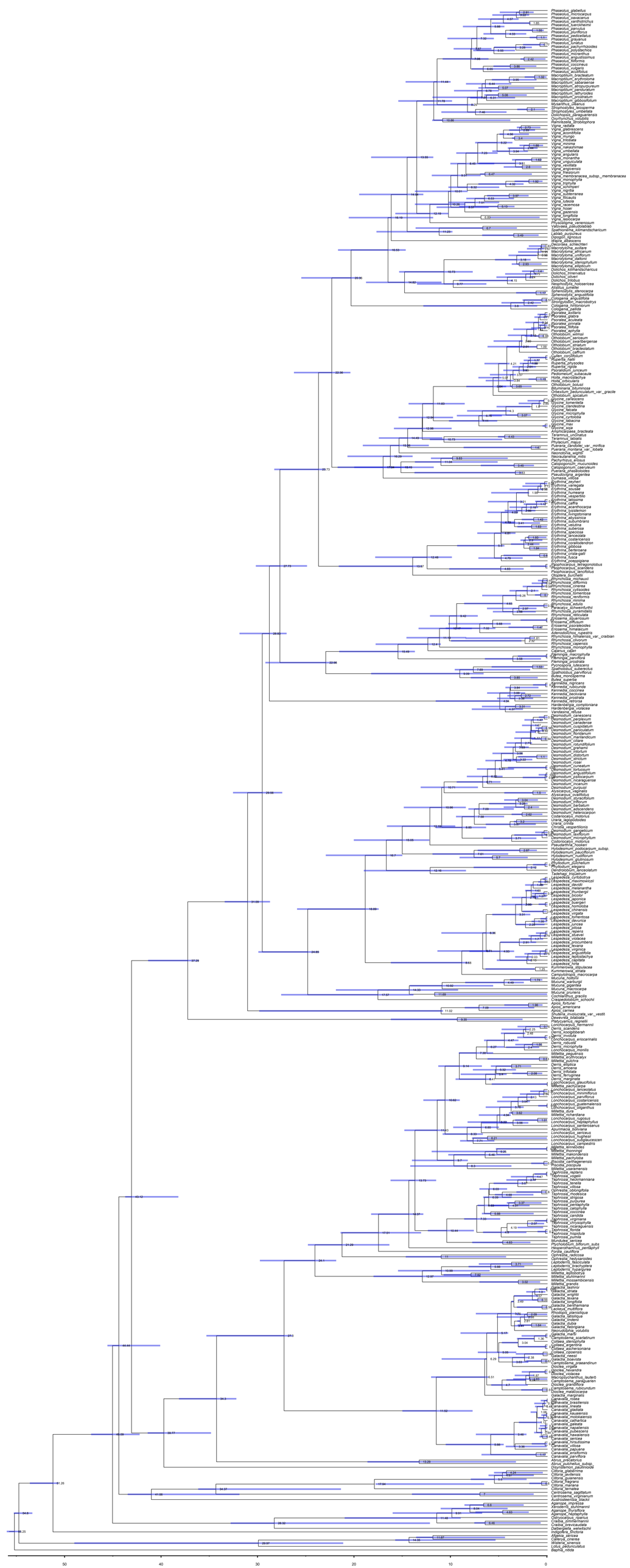

Supplement: Supplementary file 1 [file genes-13-02220-s001.zip › Figure S3. Chronogram for the millettiod legumes obtained under a Bayesian relaxed clock.pdf]

Figure 4-4B

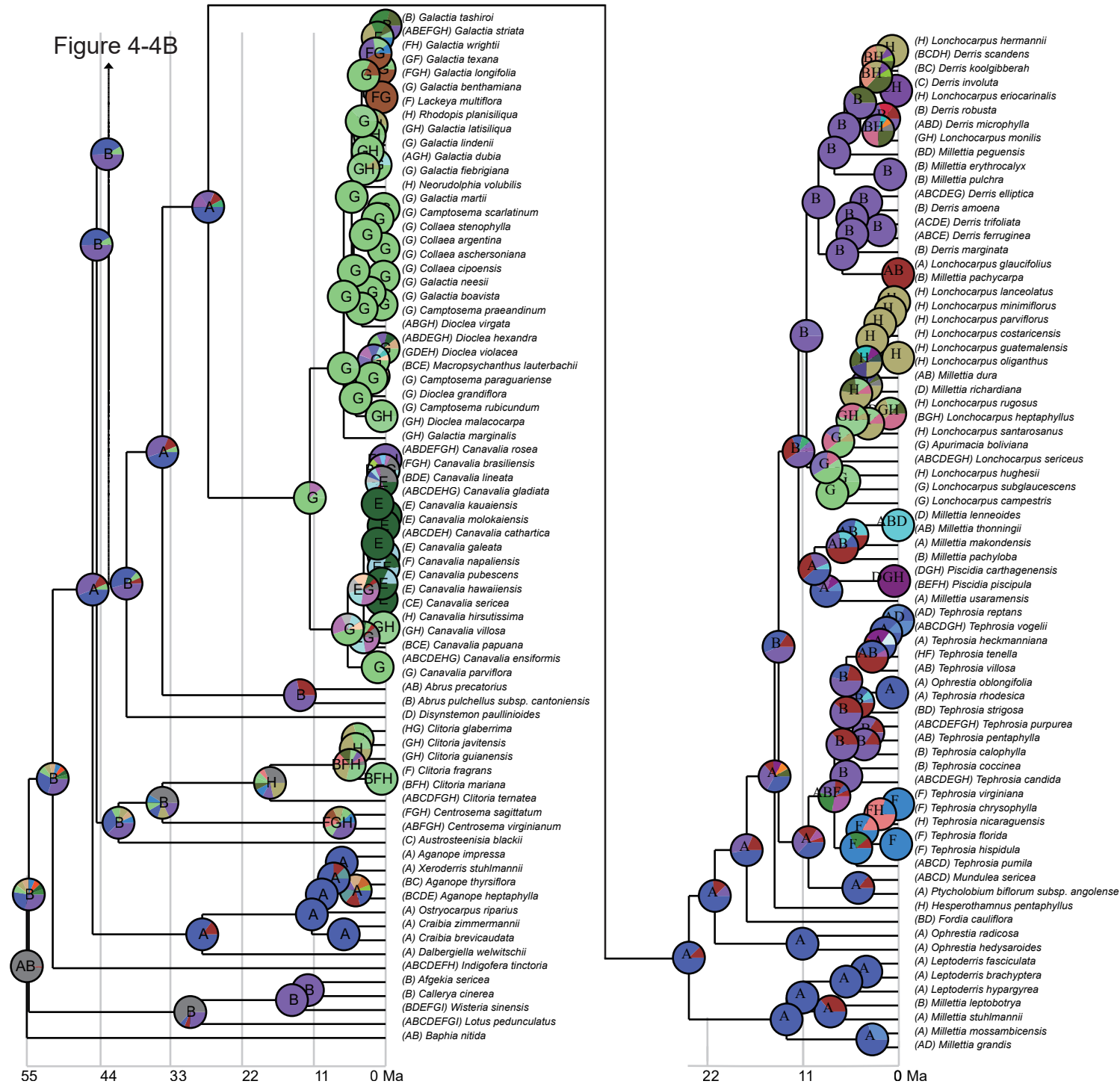

Figure 4-4A

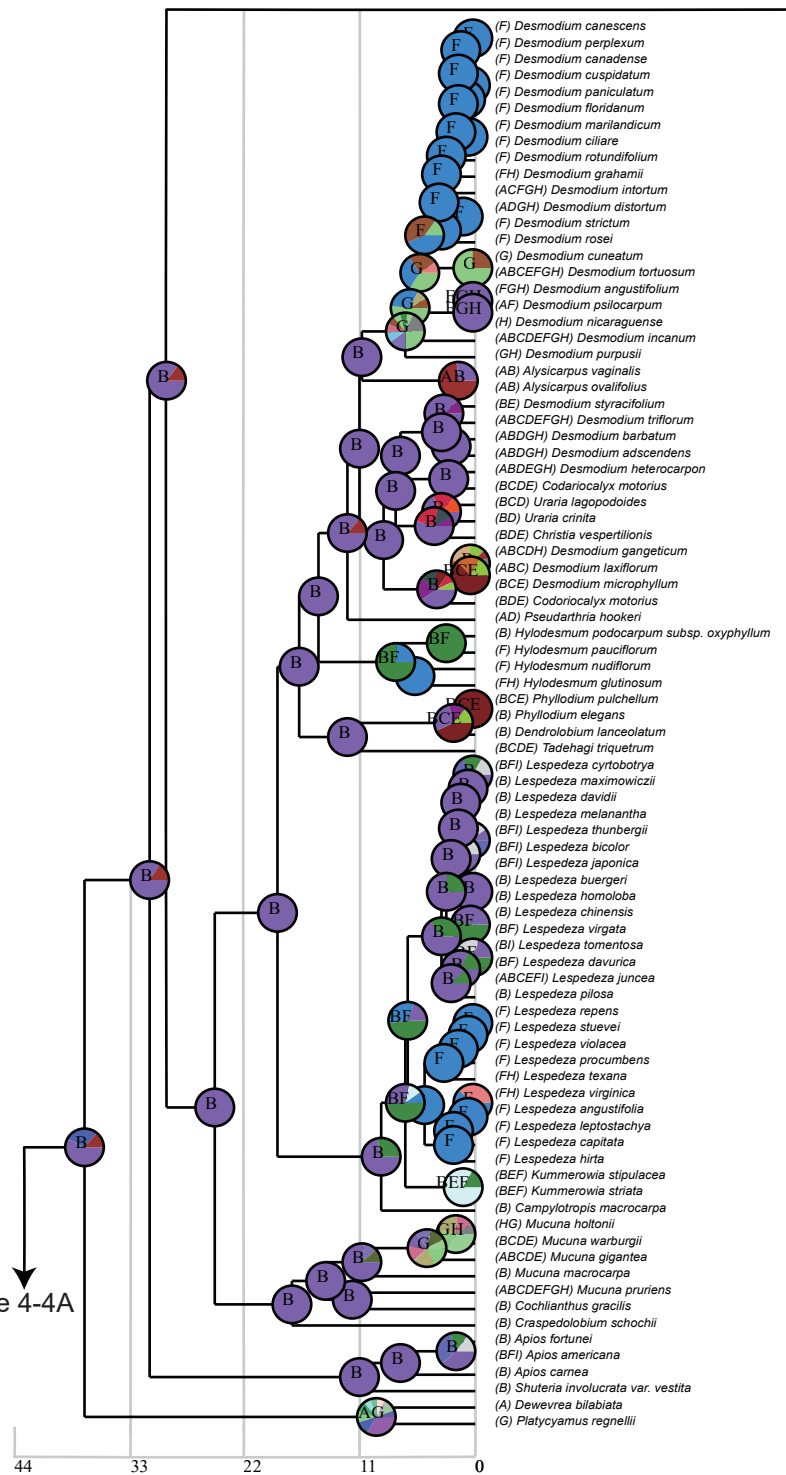

Figure 4-4C

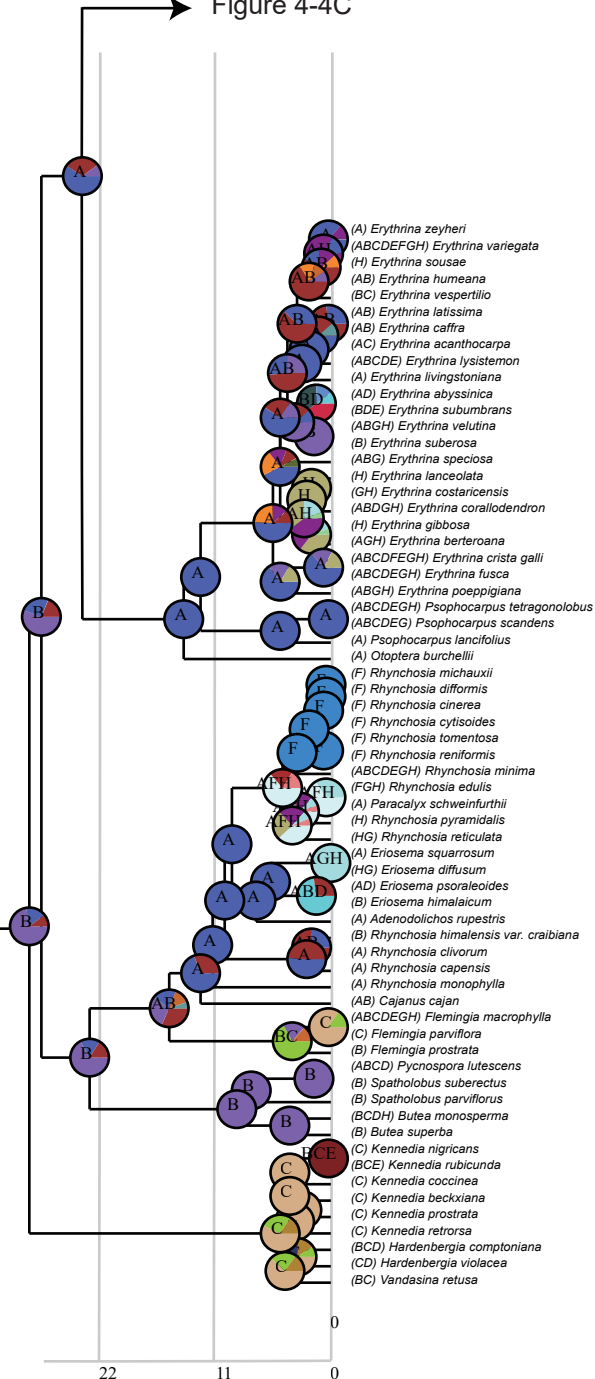

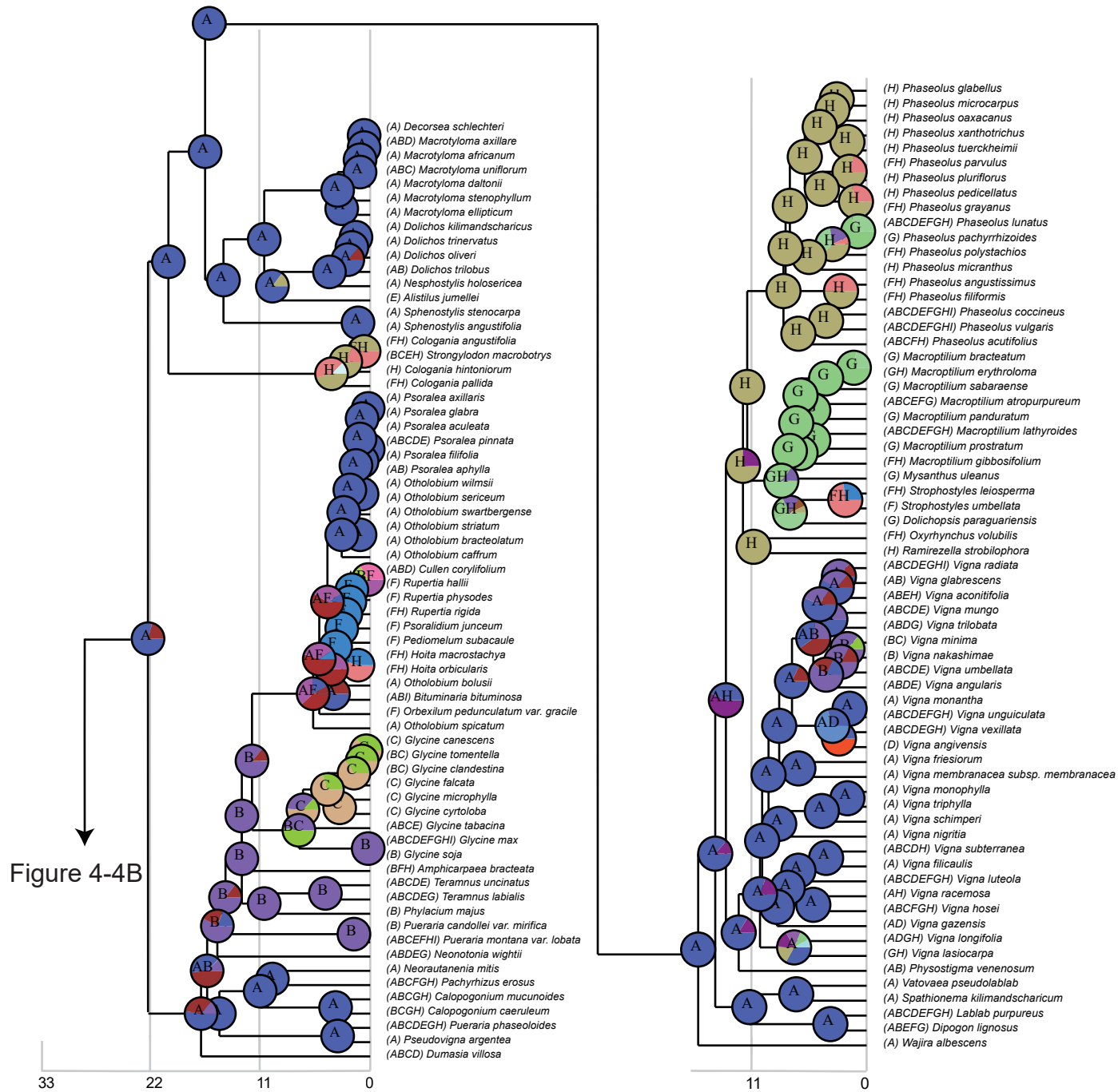

Supplement: Supplementary file 1 [file genes-13-02220-s001.zip › Figure S4. Biogeographic analysis of the millettiod legumes by Statistical Dispersal-Vicariance Analysis (S-DIVA).pdf]
