# Supplementary material for: Interplay of Ecological Opportunities and Functional Traits Drives the Evolution and Diversification of Millettiod Legumes (Fabaceae)
Source: Genes (Basel). 2022 Nov 27;13(12):2220. doi: 10.3390/genes13122220 (PMC9777679; doi:10.3390/genes13122220)
Supplement: Supplementary file 1 [file Figure S2. Phylogenetic relationships of the phaseoloid legumes using Bayesian inference (BI) method. Numbers near nodes are posterior probabiliti.pdf › Figure S2. Phylogenetic relationships of the phaseoloid legumes using Bayesian inference (BI) method. Numbers near nodes are posterior probabiliti.pdf]

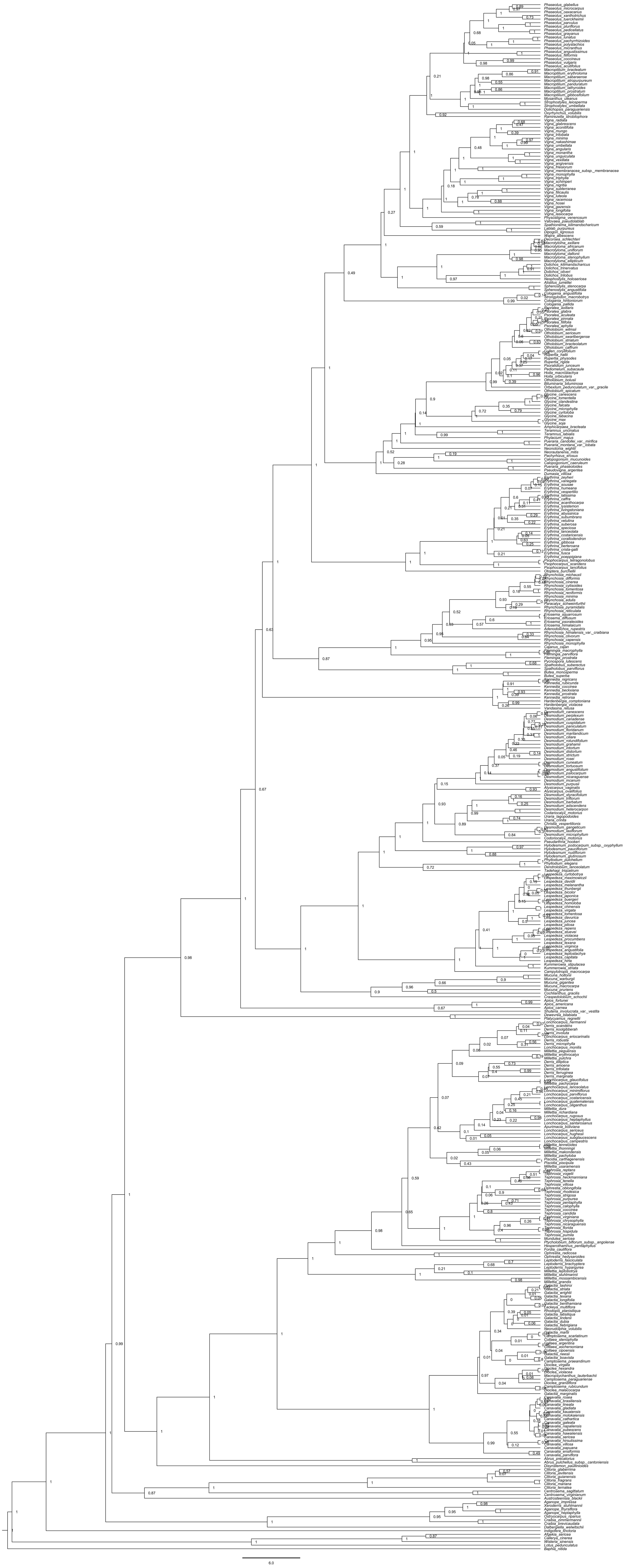
6.0
